# Functional Insights in PLS3-Mediated Osteogenic Regulation

**DOI:** 10.3390/cells13171507

**Published:** 2024-09-09

**Authors:** Wenchao Zhong, Janine Neugebauer, Janak L. Pathak, Xingyang Li, Gerard Pals, M. Carola Zillikens, Elisabeth M. W. Eekhoff, Nathalie Bravenboer, Qingbin Zhang, Matthias Hammerschmidt, Brunhilde Wirth, Dimitra Micha

**Affiliations:** 1 Department of Human Genetics, Amsterdam UMC Location Vrije Universiteit Amsterdam, 1081 HV Amsterdam, The Netherlands; w.zhong@amsterdamumc.nl (W.Z.); g.pals@amsterdamumc.nl (G.P.); 2 Department of Clinical Chemistry, Amsterdam UMC Location Vrije Universiteit Amsterdam, 1081 HV Amsterdam, The Netherlands; n.bravenboer@amsterdamumc.nl; 3 Amsterdam Movement Sciences, Tissue Function And Regeneration, 1081 HV Amsterdam, The Netherlands; emw.eekhoff@amsterdamumc.nl; 4 Department of Temporomandibular Joint, School and Hospital of Stomatology, Guangdong Engineering Research Center of Oral Restoration and Reconstruction & Guangzhou Key Laboratory of Basic and Applied Research of Oral Regenerative Medicine, Guangzhou Medical University, Guangzhou 510060, China; j.pathak@gzhmu.edu.cn (J.L.P.); 2020686081@gzhmu.edu.cn (X.L.); qingbinzhang@gzhmu.edu.cn (Q.Z.); 5 Institute of Human Genetics University Hospital of Cologne, University of Cologne, 50931 Cologne, Germany; janine.neugebauer@currenta.biz (J.N.); brunhilde.wirth@uk-koeln.de (B.W.); 6 Center for Molecular Medicine Cologne, University of Cologne, 50931 Cologne, Germany; 7 Department of Internal Medicine, Erasmus MC, University Medical Center Rotterdam, 3015 CE Rotterdam, The Netherlands; m.c.zillikens@erasmusmc.nl; 8 Department Internal Medicine, Endocrinology and Metabolism, Amsterdam UMC Location Vrije Universiteit Amsterdam, Rare Bone Disease Center, 1081 HV Amsterdam, The Netherlands; 9 Amsterdam Reproduction and Development, 1105 AZ Amsterdam, The Netherlands; 10 Developmental Biology Unit, Institute of Zoology, University of Cologne, 50931 Cologne, Germany; mhammers@uni-koeln.de; 11 Center for Rare Diseases, University Hospital of Cologne, University of Cologne, 50931 Cologne, Germany

**Keywords:** plastin-3, osteogenic differentiation, transcriptome, osteoporosis, actin-bundling protein

## Abstract

Plastin-3 (PLS3) encodes T-plastin, an actin-bundling protein mediating the formation of actin filaments by which numerous cellular processes are regulated. Loss-of-function genetic defects in PLS3 are reported to cause X-linked osteoporosis and childhood-onset fractures. However, the molecular etiology of PLS3 remains elusive. Functional compensation by actin-bundling proteins ACTN1, ACTN4, and FSCN1 was investigated in zebrafish following morpholino-mediated *pls3* knockdown. Primary dermal fibroblasts from six patients with a *PLS3* variant were also used to examine expression of these proteins during osteogenic differentiation. In addition, *Pls3* knockdown in the murine MLO-Y4 cell line was employed to provide insights in global gene expression. Our results showed that ACTN1 and ACTN4 can rescue the skeletal deformities in zebrafish after *pls3* knockdown, but this was inadequate for FSCN1. Patients’ fibroblasts showed the same osteogenic transdifferentiation ability as healthy donors. RNA-seq results showed differential expression in *Wnt1*, *Nos1ap*, and *Myh3* after *Pls3* knockdown in MLO-Y4 cells, which were also associated with the *Wnt* and *Th17* cell differentiation pathways. Moreover, WNT2 was significantly increased in patient osteoblast-like cells compared to healthy donors. Altogether, our findings in different bone cell types indicate that the mechanism of PLS3-related pathology extends beyond actin-bundling proteins, implicating broader pathways of bone metabolism.

## 1. Introduction

In 2013, we demonstrated that variants in *PLS3* (plastin 3, also known as T-plastin or fimbrin) are an X-linked genetic cause of nonsyndromic osteoporosis [1]. Male patients present severe osteoporosis and fractures from a young age, while the clinical presentation in females is variable [2]. It is estimated that 65% of published cases are men, of whom around 70% are diagnosed before the age of 18 [3]. Disease manifestations include in particular peripheral fractures and vertebral compression fractures, in the presence of low bone mineral density (BMD), and low bone turnover rate. *PLS3* pathogenic variants lead to loss-of-function, and currently, more than 27 have been reported, including frameshift, nonsense, and splice site defects as well as large intragenic deletions and duplications. Yet, no correlations have been identified between genetic defect types and the BMD score or other bone properties [4]. Even though PLS3 was originally reported as a form of nonsyndromic osteoporosis, some patients are known to have additional clinical features such as developmental delay [5], or neuromuscular abnormalities, like waddling gait [6]. Intriguingly, some patients also exhibit extraskeletal symptoms reminiscent of osteogenesis imperfecta (OI), including translucent teeth, blue sclera, and joint hypermobility [1,7].

Due to the complex function of PLS3, the pathogenic mechanism leading to osteoporosis and fractures has remained largely elusive. PLS3 is important for diverse cell functions because it is involved in F-actin binding and bundling, facilitating cell processes such as motility, focal adhesion, cell division, endocytosis, neurotransmission, vesicle trafficking, axonal local translation, and intracellular calcium regulation [3]. Based on this, it is largely unclear how PLS3 can influence the function and communication of bone cell types, and bone matrix composition. Given that PLS3 shows a wide pattern of expression, why only the bone tissue is affected in these patients is puzzling. Thus, elucidating the exact role of PLS3 in bone tissue, and its relationship to other tissue types, can potentially be informative regarding the disease mechanism.

Some insights in the bone-regulatory role of PLS3 have been obtained from animal models. Our previous study demonstrated the role of PLS3 in the skeletal development of zebrafish. Malformations in developing craniofacial bone structure, body axis, and tail were visible in a *col1a1:eGFP* transgenic zebrafish line following treatment with *pls3* antisense translation-blocking morpholinos (MO); this could be reversed dose-dependently by the administration of human *PLS3* mRNA [1]. In *Pls3*^tm1a(EUCOMM)Wtsi^ mice, the ubiquitous *Pls3* knockout mediated expression deficiency and reduced cortical thickness; the phenotype was established at an early postnatal stage and persisted to adult life despite normalized bone turnover markers [8]. Moreover, in the same mouse model, besides a significant decrease in cortical thickness, severely impaired trabecular bone structure, cortical area, and cortical area-to-tissue area ratio were shown [9]. In line with the above, the rat knockout model PLS3^E10−16del/0^ also confirmed the phenotype of early-onset osteoporosis, which was curable by alendronate and teriparatide treatment [10]. However, osteoclast-specific knockout of *Pls3* in mice failed to induce an osteoporotic phenotype, which indicates the complex mechanistic nature of PLS3 and the potential involvement of multiple cell types, necessitating diverse models [11]. 

To investigate the pathological mechanism of PLS3 defects in bone tissue, and the absence thereof in unaffected tissue types, we sought to investigate the possible presence of compensation mechanisms. PLS3 belongs to a superfamily of calponin homology (CH) domain-containing actin cytoskeleton organizers, and is unique in having two actin-binding domains (ABDs) as a monomer [12]. Thus, it can be hypothesized that other actin-bundling proteins with ABDs, such as α-actinins and fascin 1, may be able to compensate for the effect of PLS3 defects in unaffected tissues of the patients. In this study, we investigated whether Actinin1 (ACTN1), Actinin4 (ACTN4), and Fascin1 (FSCN1) expression can reverse the skeletal defects in a *col1a1:EGFP* transgenic zebrafish model with *pls3* morpholino (MO)-mediated knockdown. Osteoblast-like cells (OBs) generated from patients with pathogenic *PLS3* variants, were investigated for their mineralization ability, alkaline phosphatase (ALP) activity, and abundance of actin-bundling proteins and osteogenic markers. Finally, considering the potential role of PLS3 in mechanosensing, we used the murine osteocyte-like MLO-Y4 cell line to examine changes in transcriptome expression after *Pls3* knockdown. 

## 2. Materials and Methods

### 2.1. In Vivo Characterization of pls3 Knockdown Zebrafish Compensated with Actin-Bundling Proteins

The *col1a1:EGFP* transgenic line was a kind gift from Shannon Fisher to Matthias Hammerschmidt’s lab. Adult zebrafish and embryos were kept at ~28 °C and maintained by standard protocols [13]. Antisense MO injection and visualization of skeletal deformities were performed as previously described [1]. To investigate the specificity of the *pls3* MO effect, rescue experiments were performed: in brief, human *ACTN1*, *ACTN4*, and *FSCN1* mRNA was in vitro transcribed using the mMESSAGE mMACHINE kit (Ambion, Waltham, MA, USA) and 300 pg mRNA was co-injected with the *pls3* MO. 

### 2.2. Acquisition of Human Material

In order to investigate potential compensatory proteins and osteogenic differentiation, primary dermal fibroblasts were obtained from skin biopsies of 5 healthy controls and 6 patients with pathogenic *PLS3* variants (Table 1). The gender of the healthy controls was variable, with males (*n* = 3) and females (*n* = 2), while the age varied from 4 to 54 years old. The patients with pathogenic variants in *PLS3* were male while their age varied from 6 to 54 years old. Dermal fibroblasts were isolated and cultured from a 3 mm full-thickness skin biopsy using methods previously described [14]. Permission was granted by the local medical ethics committee (METc VUmc) and all experiments were performed in accordance with local guidelines and regulations. Informed consent was obtained from all participants and/or their legal guardians. All primary fibroblast cultures used in this study tested negative for mycoplasma.

### 2.3. Cell Culture and Osteogenic Transdifferentiation

Primary human dermal fibroblasts obtained from skin biopsies were cultured in Ham’s F-10 Nutrient Mix medium (Gibco BRL) supplemented with heat-inactivated 10% fetal bovine serum (FBS, Gibco BRL) and 1% penicillin/streptomycin (Gibco BRL, Life Technologies, Ltd., Paisley, UK). The osteogenic differentiation was performed in 48-well plates (Cellstar^®^, Greiner Bio-One GmbH, Frickenhausen, Germany) with a density of 25,000 cells per well after overnight cell attachment using the following conditions: a. Fibroblast medium consisting of the same culture medium as above. b. Osteogenic medium of minimum essential medium alpha (α-MEM) (Gibco BRL), 90 µg/mL L-ascorbic acid-2-phosphate (Sigma Aldrich Chemie NV, Zwijndrecht, The Netherlands), 5 mM β-glycerol-phosphate (Sigma Aldrich Chemie NV), 0.2% heparin (Leo Pharma, Ballerup, Denmark), 5% human platelet lysate (Stemcell Technologies, Vancouver, BC, Canada), and 1% penicillin/streptomycin (Gibco BRL). Osteogenic medium was freshly made, and it was replaced every 3 days during the osteogenic transdifferentiation time course. The MLO-Y4 cell line was cultured in α-MEM enriched with glutaMAX mix (Gibco), supplemented with heat-inactivated 2.5% FBS, 2.5% calf serum (HyClone, Logan, UT, USA), and 1% penicillin/streptomycin. All cell lines for this study were cultured at 37 °C and 5% CO_2_ in a humidified atmosphere.

### 2.4. RNA Isolation, cDNA Synthesis, and qPCR

RNA was isolated with Quick-RNA MiniPrep (Zymo Research, Irvine, CA, USA) according to the manufacturer’s instructions. The quality and quantity of RNA were measured with NanoDrop 1000 (Thermo Fisher Scientific Inc., Cleveland, OH, USA). The cDNA was constructed with Reverse Transcriptase II (Thermo Fisher Scientific Inc.) according to the manufacturer’s protocol. Expression of *PLS3*, *ACTN1*, *ACTN4*, *FSCN1*, and osteoblast markers was measured with qPCR performed in 384-well plates using the LightCycler^®^ 480 (Roche Diagnostics, Basel, Switzerland) with the following PCR program: pre-denaturation at 95 °C for 2 min followed by 45 cycles of denaturation at 95 °C for 10 s, annealing at 60 °C for 20 s, and extension at 72 °C for 30 s. The primers (Invitrogen, Thermo Fisher Scientific, Cleveland, OH, USA) were designed with the software program LightScanner Primer Design version number: 1.0 (Idaho Technology Inc., Salt Lake City, UT, USA) and are listed in Table 2.

All samples were analyzed in duplicate using LightCycler 480 Software 1.5.0 SP4 (Roche Diagnostics, Basel, Switzerland). The mRNA levels of the target transcripts were normalized to the housekeeping *GAPDH* or *Tbp* mRNA using advanced relative quantification analysis. 

### 2.5. Western Blotting Analysis

Primary fibroblasts, transdifferentiated fibroblasts, osteoblast-like cells, and MLO-Y4 cells were lysed in NuPAGE^®^ LDS Sample Buffer with NuPAGE^®^ reducing agent by scraping. The whole-cell lysates were subjected to electrophoresis, after which proteins were transferred to a nitrocellulose membrane with the iBLOT transfer system (Invitrogen). After incubation in blocking buffer for 1 h (LI-COR Biosciences, Lincoln, NE, USA), the nitrocellulose membrane was incubated with the primary antibodies overnight at 4 °C. Visualization of the signal was performed after incubation with the secondary antibodies IRDye 800 CW goat anti-rabbit IgG and IRDye 680 CW goat anti-mouse IgG antibodies by using the Odyssey infrared imaging system equipped with the Odyssey version 4 software program (LI-COR Biosciences). The relative protein expression was quantified using Image Studio Lite (LI-COR Biosciences). The primary antibodies include PLS3 (1:1000, Eurogentec, Seraing, Belgium; Cat#13013), ACTN1 (1:500, Abcam, Cambridge, UK; Cat#ab68194), ACTN4 (1:1000, Abcam; Cat#ab108198), FSCN1 (1:500, Eurogentec; Cat#ZNL1313143), and TUBA4A (1:2000, Abcam; Cat#ab7291).

### 2.6. Pls3 Knockdown in Murine MLO-Y4 Cell Line

For each 6-well, 150 µL of 6% Lipoofectamine RNAiMAX (Invitrogen) diluted in Opti-MEM (Gibco Life Technologies) was used. Small interfering RNA-*Pls3* (si-*Pls3*) (Ambion, Cat#s10689) and si-control (Ambion, Cat#4390843) were diluted in Opti-MEM to a concentration of 200 nM and then 1:1 with the diluted RNAiMAX to a final concentration of 100 nM of siRNA, non-targeting siRNA (negative control) or no siRNA (untreated). The mix was incubated at room temperature for 5 min. After that, the cells were washed with 2 mL 1 × HBSS (Gibco). A quantity of 250 µL of the lipofectamine/siRNA mixture was added to each well, followed by 10 min incubation at room temperature. For RNA sequencing, 200,000 MLO-Y4 cells were seeded per Poly L-lysine-coated glass slide (25.4 mm × 76.2 mm, Fisherfinest, Hampton, NH, USA) and 750 µL of the lipofectamine/siRNA mixture was added to each slide followed by 10 min incubation at room temperature.

### 2.7. RNA Extraction, cDNA Library Establishment, and Illumina Sequencing

Total RNA was extracted from MLO-Y4 cells using TRIzol reagent (Invitrogen) at 24 h after si-*Pls3* transfection. Agarose gel (1%) was used to monitor RNA degradation and contamination. The purity of RNA was checked using a NanoPhotometer spectrophotometer (Implen). The RNA concentration was measured using the Qubit RNA assay kit in the Qubit 2.0 Fluorometer (Life Technologies, Carlsbad, CA, USA). The RNA integrity was evaluated using the RNA Nano 6000 assay kit of the Bioanalyzer 2100 System (Agilent Technologies, Santa Clara, CA, USA). Polyadenylated RNA-seq was performed using standard Illumina Truseq kits (NEB, Ipswich, MA, USA), and samples were sequenced using the Illumina HiSeq 4000 PE150.

### 2.8. RNA Sequence Data Analysis

Low-quality base and adapters were removed by using “fastp” [15]. Clean reads were aligned to the mouse genome (mm10) reference genome using HISAT [16]. HTSeq was used to count the number of reads mapped to each gene [17]. The calculation of gene expression followed the FPKM method (Fragment Per kb per Million Fragments) and RPM method (back-spliced reads per million mapped reads), respectively. The KEGG database was used to analyze the enriched genes. KEGG analysis was performed using the “clusterProfiler” R package [18]. KEGG terms with corrected *p* < 0.05 were considered significantly enriched.

### 2.9. Mineralization Assay

For alizarin red S (ARS) staining, cells were washed with 2 mL EBSS (Gibco BRL) and fixed with 10% formaldehyde for 15 min at room temperature after 28 days in culture. The cells were rinsed three times with MilliQ water followed by the incubation of cells with 200 µL/well Alizarin Staining Solution (EMD Millipore, Burlington, MA, USA) at room temperature for 10 min. The cells were then washed with MilliQ water four times for 5 min on a shaking platform. ALP staining was performed after fixation with 10% formaldehyde by incubating the cells for 10 min in 0.1 M Tris-buffered saline with pH 7.6 followed by 10 min incubation in ALP buffer with pH 10–11. The cells were incubated with BCIP/NBT Color Development Substrate (Promega, Madison, WI, USA) solution for 5 min at 37 °C for ALP staining. Deionized water was used to stop the reaction. Mineralization was quantified from the acquired images using Fiji [19]. 

### 2.10. Statistical Analysis

Chi-square test was used to assess the association between different rescue proteins in *pls3* MO knockdown zebrafish and its deformity. A One-way ANOVA measurement, corrected by a Tukey test, was performed to determine if there was a significant difference expression of the different bone-specific markers in cells treated with fibroblast medium and osteogenic differentiation medium. Unpaired test was used to test differences in gene expression measured by RT-qPCR between controls and patients, and RNA-seq between si-control and si-*Pls3* MLO-Y4 cell line. Differences were considered significant if *p* < 0.05. All analyses were performed using GraphPad Prism software 9 (GraphPad, San Diego, CA, USA).

## 3. Results

### 3.1. Actinin-1 and Actinin-4 Rescue Malformations of Craniofacial Bone Structure, Body Axis, and Tail in 5-Day-Old col1a1-eGFP Zebrafish with pls3-Mediated Morpholino Knockdown

**Figure 1:**
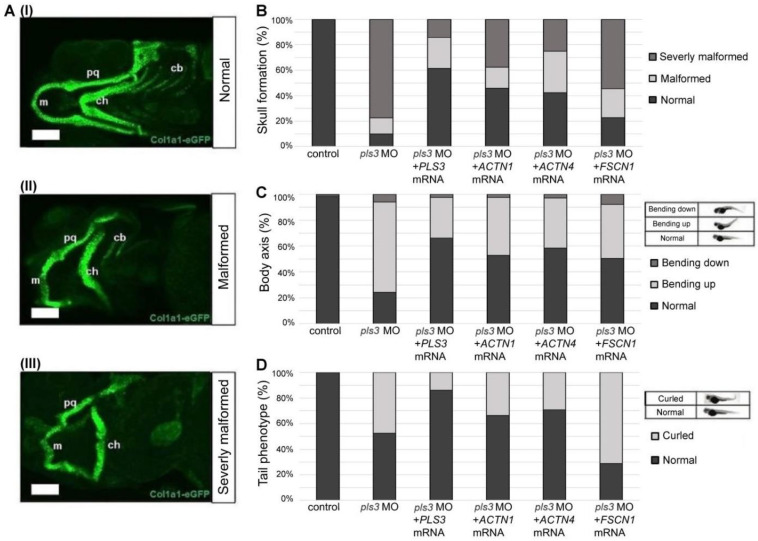
Actinin-1 and Actinin-4 rescue malformation of craniofacial bone structure, body axis, and tail in 5-day-old col1a1:eGFP zebrafish with *pls3* morpholino (*pls3* MO). (**A**) Overview of different observed phenotypes of developing skeletal elements in col1α1:eGFP fish (cartilage green) with and without *pls3* MO knockdown. Phenotype is subdivided into three different types of severity: (**I**) normal: all structures are present and developed normally; (**II**) malformed: m, ch, and pq are shorter and partially malformed; (**III**) severely malformed: m, ch, and pq are shorter and partially malformed, some cb are absent. (m: Meckel’s cartilage; ch: ceratohyal; pq: palatoquadrate; cb: ceratobranchial). (**B**) Statistical analysis of phenotypes of control fish compared to knockdown and rescue fish. Control fish show 100% normal skull development, which is significantly reduced to 10.11% in 0.8 mM *pls3* MO-treated fish (*p* < 0.0001). This effect is rescued by co-injection of 300 pg human *PLS3*, *ACTN1*, and *ACTN4* mRNA (61.43%, 45.90%, and 42.30% normal skull, respectively, compared to *pls3* MO: *p* < 0.0001). Co-injection of *FSCN1* mRNA showed 22.72% normal skull development compared to *pls3* MO: *p* = 0.1105. (**C**) Analysis of body axis form with exemplary pictures for categorization. Control fish show 98.44% normal body axis, which is significantly reduced to 24.32% in 8 mM *pls3* MO-treated fish (*p* < 0.0001). This effect is rescued by co-injection of 300 pg human *PLS3*, *ACTN1*, *ACTN4*, and *FSCN1* mRNA (66.28%, 52.87%, 58.47%, and 50.55% normal body axis, respectively, compared to *pls3* MO: *p* < 0.0001). (**D**) Analysis of tail form with exemplary pictures for categorization. Control fish show 100% normal tail, which is significantly reduced to 52.43% in 0.8 mM *pls3* MO-treated fish (*p* < 0.0001). This effect is rescued by co-injection of 300 pg human *PLS3, ACTN1*, and *ACTN4* mRNA (86.05%, 65.52%, and 72.68% normal tail, respectively, compared to *pls3* MO: *p* < 0.0001, *p* < 0.05, *p* < 0.001, and *p* < 0.001). Co-injection of FSCN1 mRNA only showed 31.87% normal tail compared to *pls3* MO: *p* < 0.001 (for every experimental set-up, n > 50; scale bar = 100 μm).

To understand whether actin-bundling proteins play an important role in compensating PLS3 function, pls3 MO-treated col1α1a:eGFP zebrafish were co-injected with 300 pg human *ACTN1*, *ACTN4*, or *FSCN1* mRNA. Histological analysis showed defects in skull formation, body axis, and tail phenotype following *pls3* MO knockdown (Figure 1). Interestingly, in almost 35% and 32% of the abnormal fish, skull formation was rescued after adding *ACTN1* and *ACTN4*, respectively (Figure 1B). In 76% of *pls3* knockdown zebrafish with defective body axis, co-injection of *ACTN1* or *ACTN4* provided 28% and 34% rescue, respectively (Figure 1C). In addition, 48% *pls3* of knockdown fish had curled tail phenotype, which decreased to 33% and 30%, respectively, after *ACTN1* and *ACTN4* mRNA treatment (Figure 1D). However, after co-injection of 300 pg human *FSCN1* mRNA, only body axis rescue was significantly improved compared to the MO-treated fish (Figure 1B–D).

### 3.2. PLS3 Expression in Fibroblasts of PLS3-Variant Osteoporosis Patients

**Figure 2:**
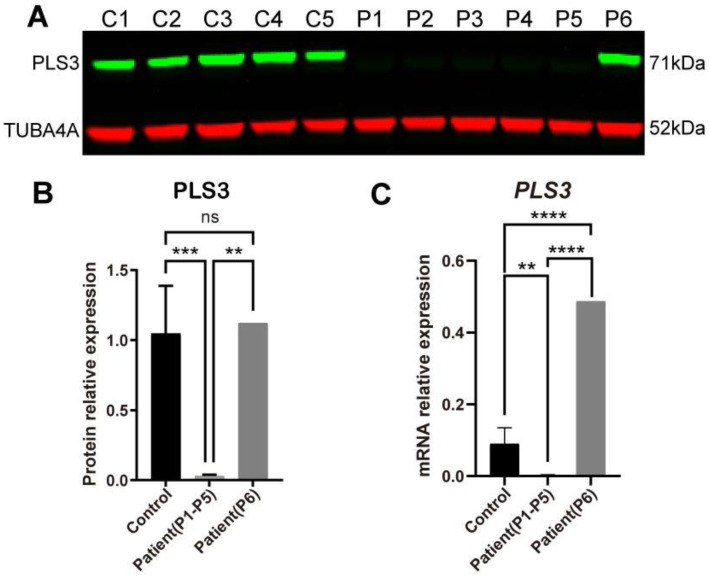
PLS3 expression in fibroblasts from healthy donors and PLS3 patients. (**A**) PLS3 is undetectable in fibroblast lysates of patients 1(P1), P2, P3, and P4 with the c.235del p.(Tyr79fs) frameshift mutation and P5 with the c.748+1G→A mutation. PLS3 expression of P6 with c.759_760insAAT insertion is stable. (**B**) Quantified Western blot results. (**C**) Relative gene expression of *PLS3* was measured by qPCR; GAPDH was used to normalize gene expression (error bars indicate standard deviation, ** *p* < 0.01, *** *p* < 0.001, **** *p* < 0.0001).

The mRNA expression and protein abundance level of PLS3 was investigated in dermal fibroblasts of six osteoporosis patients with pathogenic *PLS3* variants and five healthy individuals [1] (Table 1). Four patients from the same family (P1–P4) had the (c.235del p.(Tyr79fs)) frameshift variant. Patient 5 (P5) had the splice-site variant c.748+1G→A in exon 7; patient 6 (P6) had the insertion variant c.759_760insAAT; p.(Ala253_Leu254insAsn) in exon 8 (Table 1). PLS3 protein abundance in patients with *PLS3* variants (named from now on PLS3 patients) was compared to healthy individuals by Western blot analysis (Figure 2A). In agreement with the predicted effect of the variant, significantly lower levels in PLS3 protein were detected in the P1–P5 fibroblasts (*p* < 0.001) but not P6 (Figure 2B). The mRNA expression of *PLS3* was also significantly lower in the patient group P1–P5 (*p* < 0.01) but not P6 (Figure 2C).

### 3.3. Actin-Bundling Protein Expression in PLS3-Variant Patient Fibroblasts and Osteoblast-like Cells

**Figure 3:**
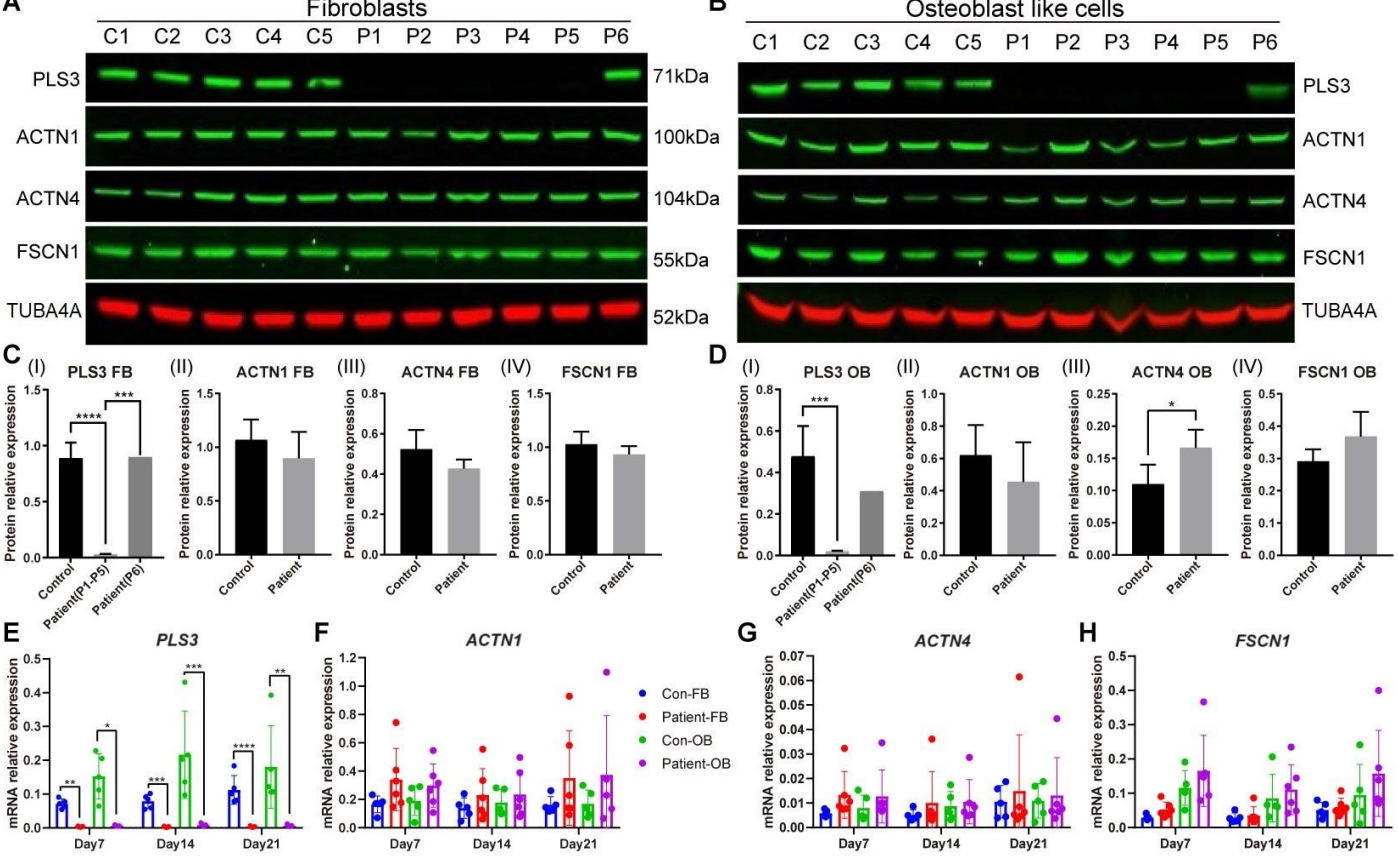
Actin-bundling protein expression in fibroblasts and osteoblast-like cells. The expression of PLS3, ACTN1, ACTN4, and FSCN1 was measured in cell lines from 5 healthy donors and 6 PLS3-variant patients on day 21. Protein expression was detected in whole-cell lysates of (**A**) primary fibroblasts (FB) and (**B**) osteoblast-like cells (OB) by Western blotting. (**C**,**D**) Quantification of Western blot results; the PLS3 expression of P6 is indicated separately. (**E**–**H**) Relative gene expression was measured by qPCR and results were normalized based on the expression of GAPDH (error bars indicate standard deviation per group, * *p* < 0.05, ** *p* < 0.01, *** *p* < 0.001, **** *p* < 0.0001).

In order to explore whether other actin-bundling proteins can potentially compensate for the function of the PLS3 protein, we measured the gene and protein expression level of ACTN1, ACTN4, and FSCN1, three actin-bundling proteins with structural and functional homology to PLS3, on day 7, 14, and 21 of osteogenic transdifferentiation (Figure 3). Although on day 21 the protein expression of ACTN1, ACTN4, and FSCN1 showed a pattern of decreased protein expression in patient fibroblasts (Figure 3C), a significant increase in ACTN4 and an increased trend of FSCN1 was observed in patient OBs (Figure 3D). When fibroblasts were stimulated with osteogenic media, no difference was observed in *ACTN1* and *ACTN4* mRNA levels between controls and patients (Figure 3F,G). However, a trend of increased *FSCN1* mRNA expression in OBs was visible in both controls and patients, with a higher increase in the patient group (Figure 3H).

### 3.4. Pls3 Knockdown in MLO-Y4 Cells

**Figure 4:**
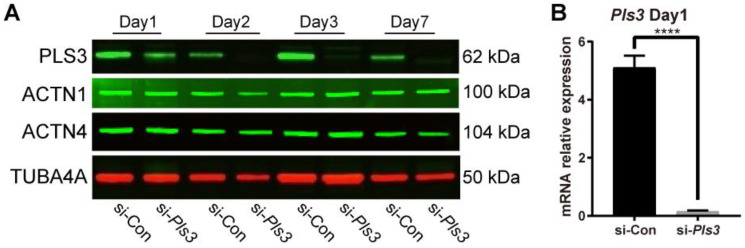
PLS3 knockdown in MLO-Y4 cells. (**A**) PLS3, ACTN1, and ACTN4 expression determined by Western blotting analysis from day 1 to 7 after the transfection. TUBA4A was used as a loading control. (**B**) *Pls3* mRNA expression after 24 h of transfection. *Tbp* was used to normalize gene expression. si-*Pls3*: small interfering *Pls3*. (Error bars indicate standard deviation, **** *p* < 0.0001).

Knockdown of *Pls3* in MLO-Y4 cells by using siRNA was used to investigate the role of PLS3 in osteocyte function. The efficacy of *Pls3* knockdown was confirmed by Western blot and qRT-PCR. Complete knockdown of *Pls3* mRNA in MLO-Y4 cells took place after 24 h transfection by siRNA (Figure 4B). PLS3 protein expression was downregulated on day 1 with a complete knockdown on days 2, 3, and 7 (Figure 4A). Interestingly, no significant differences were found in ACTN1 and ACTN4 protein expression during the time course until day 7 (Figure 4B). 

### 3.5. Differential Gene Expression in MLO-Y4 Cells Following Pls3 Knockdown 

**Figure 5:**
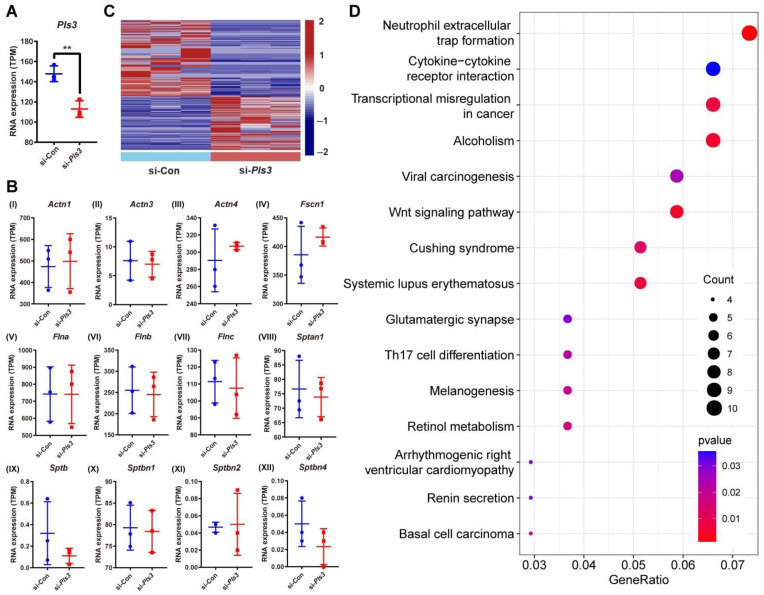
Differential gene expression in MLO-Y4 cells following *Pls3* knockdown. (**A**) Expression of *Pls3* analyzed by RNA sequencing. Significant effect of the treatment: ** *p* < 0.01. (**B**) The expression of actin-binding proteins was analyzed by RNA sequencing. (**C**) Heat map shows the expression pattern of mRNAs between the si-Con and si-*Pls3* groups: differentially upregulated mRNAs (total 259), and differentially downregulated mRNAs (total 368). (**D**) KEGG pathway enrichment analysis based on mRNA expression differences between the si-Con and si-Pls3 groups.

MLO-Y4 cells were analyzed by transcriptome sequencing following *Pls3* knockdown after siRNA treatment for 1 day; three biological replicates were included per treated and untreated (control) samples. According to the transcriptome sequencing data, *Pls3* expression was significantly decreased after siRNA treatment (Figure 5A). This is in accordance with the results of the qPCR analysis (Figure 5C) and confirms the validity of our transcriptome sequencing study. In order to identify the differentially expressed mRNAs in the *Pls3* knockdown group, the differential expression was calculated compared to the control group using the “DESeq2” R package [20]; a *q* value < 0.05 was the standard for differential transcriptome expression. In total, 259 transcripts were differentially upregulated in the untreated MLO-Y4 cells compared to the *Pls3* knockdown cells (Figure 5C, Supplementary Table S1). Similarly, a total of 368 mRNAs were differentially downregulated in the control cells compared to the *Pls3* knockdown cells (Figure 5C, Supplementary Table S1). KEGG pathway enrichment analysis of these differentially expressed mRNAs in the R package “Cluster Profiler” showed that the differentially expressed mRNAs are distributed across many signaling pathways, in relation to bone homeostasis, e.g., the *Wnt* and *Th17* cell differentiation signaling pathways (Figure 5D). No changes were identified in the mRNA expression of actin-bundling proteins (Figure 5B), in line with mRNA expression measurements by qPCR in human cells (Figure 3E–H).

### 3.6. Osteogenic Transdifferentiation Potential of PLS3-Variant Patient Fibroblasts 

**Figure 6:**
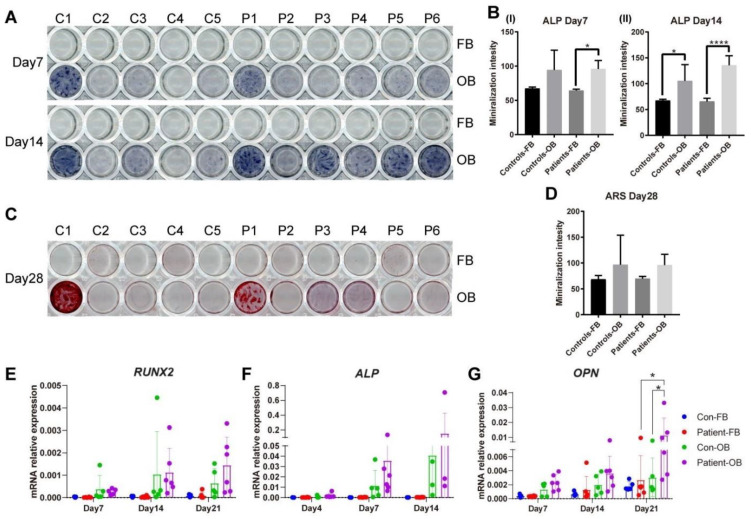
Osteogenic transdifferentiation potential of PLS3 patient-variant fibroblasts. Primary fibroblasts from healthy donors and PLS3-variant patients were subjected to osteogenic transdifferentiation (OB); undifferentiated fibroblasts (FB) were grown in fibroblast medium. (**A**) Photos show representative results for stainings performed in 11 primary fibroblast cell lines and their transdifferentiated counterparts (C1–C5, P1–P6). Positive ALP staining (purple) on day 14 indicates ALP activity. (**B**) Quantification of the intensity of ALP staining. Bars indicate the mean of the staining and error bars the standard deviation. (**C**) ARS staining (red) on day 28 indicates calcium phosphate deposits. (**D**) Quantification of the intensity of ARS staining. (**E**–**G**) Relative gene expression was measured by qPCR for (**E**) *RUNX2*, (**F**) *ALP*, and (**G**) *OPN*. *GAPDH* was used to normalize gene expression (* *p*  <  0.05, **** *p*  <  0.0001 as measured by ANOVA).

To determine the effect of PLS3 defects on osteoblast differentiation and function, transdifferentiation of fibroblasts to OBs was performed for controls and patients. For this purpose, the mineralization capacity of the transdifferentiated OBs was investigated on functional level by alizarin red S (ARS) staining, alkaline phosphatase (ALP) staining, and expression level of osteogenic markers *RUNX2*, *ALP*, and *OPN*. After treatment with osteogenic medium, both control and patient OBs showed a marked increase in ALP production after 14 days (Figure 6A), which displayed variability in intensity between different individuals per group. Likewise, ARS staining showed the same trend on day 28 (Figure 6C). After 28 days of transdifferentiation, patient OBs differed from controls by a higher level of mineralization (Figure 6C). Quantification of the stainings showed a trend of higher ALP on days 7 and 14 and higher ARS on day 28 in patient and control cells after osteogenic induction (Figure 6B,D). After transdifferentiation in osteogenic media, OBs showed a steady increase in the gene expression of osteogenic markers *RUNX2*, *ALP*, and *OPN* from day 7 compared to undifferentiated primary fibroblasts starting (Figure 6E–G); these markers are known to be upregulated during the differentiation of mesenchymal stem cells (MSCs) to osteoblasts. Even though a pattern of increased expression was observed in the patient compared to the control OBs, no statistical difference was shown at any timepoint, except for OPN expression on day 21 (Figure 6G). 

### 3.7. Gene Expression of Proteins Involved in WNTSignaling

**Figure 7:**
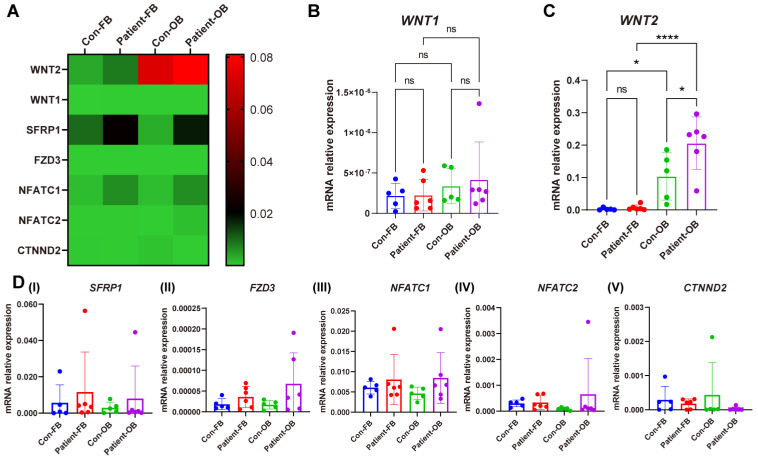
Relative gene expression of WNT pathway components in fibroblasts and osteoblast-like cells of PLS3-variant patients. Fibroblasts and osteoblast-like cells from six PLS3-variant patients and five controls. (**A**) The heat map shows the relative gene expression measured by qPCR. *GAPDH* was used to normalize gene expression. Relative gene expression of (**B**) *WNT1*, (**C**) *WNT2*, and (**D**) *SFRP1*, *FZD3*, *NFATC1*, *NFATC2*, and *CTNND2* (* *p* < 0.05, **** *p* < 0.0001).

To verify the result of KEGG pathway enrichment analysis on gene expression of the WNT pathway, the expression of seven genes that showed significant change in the WNT pathway was analyzed in fibroblasts and OBs. *WNT2* showed higher expression in patient OBs compared with control OBs and patient fibroblasts (Figure 7C). *SFRP1*, *FZD3*, and *NFATC1* showed an increasing trend in patients’ cells (Figure 7D).

## 4. Discussion

Despite the discovery of *PLS3* as a causative gene for osteoporosis over 10 years ago and the emerging of several patient families ever since, there is still a huge gap in our understanding regarding the mechanism of PLS3-induced osteoporosis [21]. Although studies have convincingly shown elevated osteoclast activity in a ubiquitous *Pls3* knockout mouse model [9], multiple cell types may be involved as shown by the absence of osteoporosis in a mouse model with osteoclast-specific *Pls3* knockout. This potentially suggests a functional interplay between different cell types, which prompted us to explore how osteoblast and osteocyte function may be affected by loss-of-function PLS3 defects in a range of diverse cell and animal models [11]. 

Owing to the structural homology of PLS3 with other actin-bundling proteins, we investigated the possibility of functional compensation in zebrafish. ACTN1 and ACTN4, similarly to PLS3, belong to the superfamily of calponin homology (CH) domain-containing actin cytoskeleton organizers with actin-binding domains (ABDs). ACTN1 and ACTN4 injection in a knockdown *pls3* zebrafish model showed strong capacity to reverse the malformation of craniofacial bone structure, body axis, and tail, similarly to *PLS3* mRNA administration. FSCN1 is also a major actin-crosslinking protein, working in concert with other crosslinkers such as α-actinin to produce filopodia [22]. However, in contrast to PLS3 and ACTNs, which can form parallel and antiparallel bundles, FSCN1 can only form parallel bundles, and differences are observed in actin filament spacing [23,24], in addition to a different ABD structure. This may explain its very limited compensatory effect for Pls3 in zebrafish. Taken together, the in vivo data confirm that Pls3 affects the formation of the zebrafish chondrocranium through its ABD protein structure with functional redundancy for ACTN1 and ACTN4, and to a much lesser extent for FSCN1. 

Despite the capacity of the actin-bundling proteins to functionally replace PLS3 in bone tissue, no increase in gene or protein expression of these actin-bundling proteins was found in fibroblasts or osteoblast-like cells of patients with loss-of-function *PLS3* pathogenic variants, resulting in either no PLS3 expression or a structural defect with the exception of ACTN4 protein in osteoblast-like cells; an increase in FSCN1 protein was not significant. The PLS3 defects also did not affect the osteogenic transdifferentiation of the cells during the first 14 days as determined by the lack of changes in RUNX2, ALP, and OPN. On day 21, patient osteoblast-like cells showed significantly higher expression of OPN compared to the control group. One potential reason is that the control group material originated from older donors compared to the patient group. PLS3 has been shown to influence mineralization, which correlates with the mechanosensory properties of osteoblasts [25]. Interestingly, lack of PLS3 protein has been shown to reduce the deposition of minerals in culture following collagen production in immortalized murine pre-osteoblast cells [8]. In our model of primary patient cells, no significant differences between patients and controls were found in alizarin red or ALP stainings at earlier timepoints. Stainings showed high variability even within cell groups, in accordance with previous studies, which may be related to the differences in the age of the donors during biopsy acquisition [14]. However, the mineralization pattern did not clearly correlate with osteogenic marker expression and thus with the state of osteogenic differentiation in the different donors. Staining heterogeneity and the requirement for more complex models facilitating interaction of different cell types [11] may explain the inability to detect hypomineralization as reported in the bone tissue of patients with PLS3 defects and in PLS3-deficient mice [6,8,26]. 

As previously stated, the ABD protein structure of PLS3 is essential in the regulation of actin dynamics in processes of cell motility, focal adhesion, cell division, endocytosis, vesicle trafficking, and motor axon outgrowth [3,27]. Based on the high expression of the PLS3 homolog fimbrin in chicken osteocyte dendrites [28] and their significance in mechanosensing [29], it is tempting to hypothesize that *PLS3* variants may compromise osteocyte mechanosensitivity and lead to osteoporosis. To investigate the effect of PLS3 loss-of-function variants in osteocytes, we used the established MLO-Y4 osteocyte-like murine cell line. Following siRNA-mediated *Pls3* knockdown for 7 days, no changes were observed in the protein expression of ACTN1 or ACTN4; FSCN1 was not possible to analyze due to the lack of a suitable antibody. Since the mechanosensing function of the *Pls3*-deficient MLO-Y4 cells was not interrogated, it remains unclear if it can be affected by decreased PLS3 expression and whether a potential functional defect could be rescued by the expression of other actin-bundling proteins. Additional studies are required to provide insights in the role of PLS3 in a setting suitable to simulate the mechanical function of osteocytes. 

Given the scarcity of studies on the mechanism of PLS3 in osteocytes, the global gene expression was investigated following *Pls3* knockdown in MLO-Y4 cells in an effort to delineate the molecular consequences of PLS3 deficiency. In line with the lack of changes in the protein expression of ACTN1 and ACTN4 after *Pls3* knockdown, no significant differences were noted in the gene expression of several actin-bundling genes. RNAseq analysis of the two cell groups revealed a total of 259 differentially upregulated mRNAs and 368 differentially downregulated mRNAs in the control cells compared to cells with *Pls3* knockdown. KEGG pathway enrichment analysis of these gene expression differences identified a role for the WNT, Th17 cell differentiation, and neutrophil extracellular traps (NETs) pathways, which are strongly related to osteoporosis. WNT signaling directly regulates bone cell development and differentiation and is a crucial component of skeletal development and homeostasis from early fetal development to pediatric and adult stage [30]. In line with this, a recent study showed that serum dickkopf-1(DKK1) concentrations were significantly elevated in *PLS3*-variant positive subjects compared with the variant-negative subjects [31]. DDK1 is an inhibitor of the WNT pathway and can bind to the transmembrane dual-receptor complex consisting of LRP5 or LRP6 and the seven transmembrane G-protein Frizzled to inactivate this receptor complex [32]. The WNT signaling cascade is also one of the most prominent pathways of bone cell mechanotransduction; the WNT co-receptor LRP5 has been identified as a crucial protein for mechanical signaling in bone [33]. Although both *PLS3* and *WNT1* genes associate with monogenic osteoporosis, there is currently no evidence of their direct mechanistic connection [34]. The TH17 cell differentiation pathway may also relate to PLS3 deficiency. TH17 cells are proinflammatory cells that release interleukin-17 (IL-17) to direct MSC differentiation towards the osteogenic lineage while indirectly increasing osteoclast differentiation [35]. As such, perturbations in this pathway could affect bone homeostasis. The NETs pathway was the most prominently affected following *Pls3* knockdown. This is the first study demonstrating a relationship between *PLS3* deficiency and NETs. Ankylosing spondylitis [36] is characterized by the higher release of NETs from polymorphonuclear leukocytes containing bioactive IL-17A and IL-1β, which can in turn mediate the osteogenic differentiation of MSCs into bone-forming cells. In contrast, NETs also reduced the viability of osteoblasts-like cells when they were stimulated by monosodium urate crystals. Supernatant obtained from osteoblast-like cells treated with NETs also promoted osteoclast differentiation [37]. More studies are needed to shed light on how the NETs may be linked to PLS3.

Among the 15 highest upregulated or downregulated differentially expressed genes, several of these are also related to bone homeostasis and bone development, such as *Klf15*, *Wnt1 Nos1ap*, and *Myh3*. Kruppel-like factor 15 (KLF15) is a key regulator of podocyte differentiation [38]; in postnatal *Pls3*-knockout mice, kidneys were macroscopically normal, but the glomerular ultrastructure showed thickening of the basement membrane and fusion of podocyte foot processes [39]. Furthermore, mapping of the amniotic fluid proteome of fetuses with congenital anomalies of the kidney and urinary tract identified PLS3 as a protein involved in glomerular integrity [39]. Further investigations may reveal if there is the possibility of crosstalk between the kidney and bone through PLS3 [40]. *WNT1* is a known gene for monogenic osteoporosis, with 35 reported loss-of-function variants affecting osteoblast differentiation and function [41]. Considering the central role of WNT1 in bone tissue regulation, the expression of 7 genes that showed significant changes in the WNT pathway was also investigated in the PLS3 mutant patient fibroblasts and OBs. PLS3 mutant patient OBs showed significantly higher *WNT2* expression than the control osteoblast-like cells on day 21 of the osteogenic transdifferentiation. This is in agreement with the osteoblast maturation during the osteogenic transdifferentiation process [42], as also shown by the upregulated *OPN* expression at this timepoint. *Nos1ap* encodes a cytosolic protein that binds to the signaling molecule, neuronal nitric oxide synthase (nNOS). nNOS plays a key role in nitric oxide production and it has a multifaceted role in bone remodeling where it is also produced after mechanical stress [43,44]. *Myh3* is also one of the most highly downregulated genes, which plays a pivotal role in fetal muscle development; defects in the human ortholog are associated with Freeman–Sheldon syndrome, distal arthrogryposis 8 (DA8), and autosomal dominant spondylocarpotarsal synostosis, which can present skeletal abnormalities, such as kyphoscoliosis and bony fusions [45]. 

## 5. Conclusions

The findings of this study support the key role of PLS3 in bone regulation through its ABD protein structure. Replacement of its ABD function by other actin-bundling proteins can reverse skeletal defects in zebrafish. However, in contrast to our expectation, fibroblasts from unaffected patient skin did not show upregulation of most actin-bundling proteins with compensatory function in the zebrafish skeleton with the exception of ACTN4. Furthermore, PLS3 deficiency in osteocyte-like cells is associated with several bone-related pathways, including WNT, TH17 cell differentiation, and NETs. In conclusion, our findings in different bone cell types indicate that the mechanism of PLS3-related pathology extends beyond actin-bundling proteins, implicating broader pathways of bone metabolism. We suspect that in vivo models or cell models that allow for functional engagement between different cell types may be key to further dissecting the mechanistic effects of PLS3 on bone fragility, which the scientific community can use to orient itself in the direction of therapeutic modalities for this severe disease.

## Figures and Tables

**Table 1:** Clinical and genetic characteristics of skin biopsy donors.

Codes	Gender	Age at Biopsy	Mutation	Mutation Type	Low-Impact Peripheral Fractures	Multiple Vertebral Fractures	Other Clinical Findings
C1	male	4	NA	NA	-	-	-
C2	male	45	NA	NA	-	-	-
C3	female	54	NA	NA	-	-	-
C4	female	45	NA	NA	-	-	-
C5	male	47	NA	NA	-	-	-
P1	male	10	PLS3: c.235del p.(Tyr79fs)	Frameshift	6	No	Acute lymphatic leukemia
P2	male	32	PLS3: c.235del p.(Tyr79fs)	Frameshift	13	Yes	None
P3	male	10	PLS3: c.235del p.(Tyr79fs)	Frameshift	Multiple	No	Epilepsy and, in childhood, waddling gait
P4	male	6	PLS3: c.235del p.(Tyr79fs)	Frameshift	17	No	Patent ductus arteriosus and, in childhood, waddling gait
P5	male	47	PLS3: c.748+1G>A	Splice site variant	Multiple	Yes	Alcohol abuse and esophageal carcinoma
P6	male	54	PLS3: c.759_760insAAT	Insertion	1	Yes	None

**Table 2:** Primers used for real-time qPCR.

Gene	Accession Number	Forward Primer (5′–3′)	Reverse Primer (5′–3′)
*PLS3*	NM_005032.4	CTCCCTGGTTGGCATTGGAG	GCCAAACTGGAGCTGATCGT
*ACTN1*	NM_001130004.1	CCCGAGCTGATTGACTACGG	ATGGCTTTCTCATCCGGTCG
*ACTN4*	NM_004924.5	GGCACAGACCAGAGCTGATT	AAGCGTGGTAGAAGCAGGAC
*FSCN1*	NM_003088.3	GTCGACTCGCTCATCACCCTC	GGAAGGTCTCCTGGTCGGTC
*RUNX2*	NM_001024630.3	ACTGCTTGCAGCCTTAAAT	ATGCTTCATTCGCCTCAC
*ALP*	NM_000478.5	AGGGACATTGACGTGATCAT	CCTGGCTCGAAGAGACC
*OPN*	NM_001040058.1	TTCCAAGTAAGTCCAACGAAAG	GTGACCAGTTCATCAGATTCAT
*SFRP1*	NM_003013.2	CGTGGGCTACAAGAAGATGG	AAGCCGAAGAACTGCATGAC
*WNT1*	NM_005430.3	GCCGATGGTGGGGTATTGTG	GATCCCCGGATTTTGGCGTA
*CTNND2*	NM_001332.3	CCGGGTGCCTAAGGAATGTT	CGCAGACTGGATCACGTACA
*WNT2*	NM_003391.2	CATCCAGATGTGATGCGTGC	GCAGATTCCCGACTACTTCG
*NFATC2*	NM_012340.4	CATGAGGGCAACCATCGACT	CCCCGTGAGGATCATTTGCT
*NFATC1*	NM_006162.4	AACCCCATCGAATGCTCCCAG	AGAAACTGACGTGAACGGGG
*FZD3*	NM_017412.3	CGCCGGGGTCTGAGATATT	GCTTGGTCTCACAAAAGGCG
*GAPDH*	NM_002046.6	GCCCAATACGACCAAATCC	AGCCACATCGCTCAGACAC
*Pls3*	NM_145629.1	CAAGCACAATAACGCCAAGTACGC	ACTCTCTTCATCCCTCTGCCCATC
*Tbp*	NM_013684.2	TGGCGGTTTGGCTAGGTTTC	CACCATGAAATAGTGATGCTGGG

## Data Availability

The data that support the findings of this study are available from the corresponding author upon reasonable request. All raw sequencing data can be accessed from the Sequence Read Archive (project accession PRJNA1106526).

## Supplementary Materials

The following supporting information can be downloaded at: https://www.mdpi.com/article/10.3390/cells13171507/s1, Table S1: mRNA significantly differential expressed in BMSCs.
